# The Failed Clinical Story of Myostatin Inhibitors against Duchenne Muscular Dystrophy: Exploring the Biology behind the Battle

**DOI:** 10.3390/cells9122657

**Published:** 2020-12-10

**Authors:** Emma Rybalka, Cara A. Timpani, Danielle A. Debruin, Ryan M. Bagaric, Dean G. Campelj, Alan Hayes

**Affiliations:** 1 Institute for Health and Sport (IHeS), Victoria University, Melbourne, Victoria 8001, Australia; danielle.debruin@live.vu.edu.au (D.A.D.); ryan.bagaric@live.vu.edu.au (R.M.B.); dean.campelj@live.vu.edu.au (D.G.C.); alan.hayes@vu.edu.au (A.H.); 2 Australian Institute for Musculoskeletal Science (AIMSS), Victoria University, St Albans, Victoria 3021, Australia; 3 Department of Medicine—Western Health, Melbourne Medical School, The University of Melbourne, Melbourne, 3021 Victoria, Australia

**Keywords:** myostatin inhibition, Duchenne Muscular Dystrophy, skeletal muscle, muscle development, clinical trials, translation

## Abstract

Myostatin inhibition therapy has held much promise for the treatment of muscle wasting disorders. This is particularly true for the fatal myopathy, Duchenne Muscular Dystrophy (DMD). Following on from promising pre-clinical data in dystrophin-deficient mice and dogs, several clinical trials were initiated in DMD patients using different modality myostatin inhibition therapies. All failed to show modification of disease course as dictated by the primary and secondary outcome measures selected: the myostatin inhibition story, thus far, is a failed clinical story. These trials have recently been extensively reviewed and reasons why pre-clinical data collected in animal models have failed to translate into clinical benefit to patients have been purported. However, the biological mechanisms underlying translational failure need to be examined to ensure future myostatin inhibitor development endeavors do not meet with the same fate. Here, we explore the biology which could explain the failed translation of myostatin inhibitors in the treatment of DMD.

## 1. Introduction

Since McPherron’s initial discovery of the mighty mouse [1] and the subsequent clinical case report of an infant with uncharacteristic muscling and superhuman strength caused by mutations in the myostatin (growth differentiation factor 8 (*GDF-8*)) gene (*MSTN*) [2], researchers and drug companies have been in a race to develop drugs targeted against myostatin protein to treat muscle wasting conditions. Therapeutic myostatin inhibition has been purported for muscular dystrophy, cachexia, sarcopenia, and disuse atrophy associated with osteoporosis, diabetes, amyotrophic lateral sclerosis and multiple sclerosis [3]. While myostatin inhibition cannot correct the primary defect in many of these diseases, severe and progressive muscle wasting could, theoretically, be attenuated, halted or reversed to increase the longevity and quality of lives of patients and reduce burden on the healthcare system. Subsequent development of a range of myostatin inhibitors and promising pre-clinical results encouraged human trials. These include: the anti-myostatin monoclonal neutralizing antibody, domagrozumab (PF-06252616) developed by Pfizer (NCT02310763); the soluble ActRIIB ligand, ACE031, developed by Acceleron Pharma, which prevents activin A, growth differentiation factor 11 (GDF-11) and some BMP ligands binding the activin A receptor 2 (ACVR2; NCT01099761); and Taldefgropbep-α (RG6206/R07239361) developed by Hoffmann-La Roche (NCT01099761), an engineered anti-myostatin adnectin™ protein. However, for Duchenne Muscular Dystrophy (DMD) (as well as several other diseases) human clinical trials have not progressed to an effective medicine.

Recently, Wagner (2020) reported on the failed clinical trials that have highlighted the futility of myostatin inhibitor drugs against DMD [4]. While “positive results bias”, a bias in part due to pharmaceutical company funded studies or because authors are more likely to submit—and journals more likely to accept—positive as opposed to negative research findings, might be influential in the failed translation of pharmacological myostatin inhibitors for DMD, the Wagner paper provided several key biological reasons for unsuccessful translation between pre-clinical and clinical studies [4]. These were: (1) the distinct differences in native myostatin levels in mice compared to humans (by ~10 fold); (2) disparity in the proportional basal suppression of circulating myostatin between wild-type/healthy and *mdx*/DMD muscles (skeletal muscle myostatin is 25% of wild-type levels in *mdx* mice compared to 8% of healthy control levels in DMD patients); and (3) the confounding effects of standard of care corticosteroid treatment in DMD patients which was never extensively tested in pre-clinical animal trials. These factors are explored in detail herein, in context of the known cellular pathophysiological events that drive DMD. We also discuss other potential factors which might alternatively explain the failed translation of myostatin inhibitor drugs, such as the important regulatory role myostatin plays on metabolism and the important role electrical stimulation plays in mechanotransduction signalling of muscle growth during myostatin inhibition, with important implications for future drug development programs.

## 2. Myostatin Is Differentially Expressed in Mice and Humans

Myostatin negatively controls skeletal muscle growth and quality through multiple molecular mechanisms. A member of the transforming growth factor β superfamily, myostatin is important for the regulation of both pre- and post-natal muscle growth. There is evidence to suggest that through interplay with GDF11, myostatin coordinates muscle growth to ensure a proportionate ratio between skeletal muscle and bone growth rate and density (as reviewed recently in [5]), such that the skeleton is capable of supporting the musculature and the musculature capable of moving the skeleton. Myostatin is a well-established inhibitor of mRNA translation, i.e., protein synthesis, in part, via targeted suppression of mammalian/mechanistic target of rapamycin complex (mTORC), a highly conserved serine/threonine kinase widely considered to be a master regulator of cell growth [6,7,8]. Additionally, myostatin drives atrophy through pro-degradative signal-transduction mechanisms in a Smad2/3-dependent manner, increasing FoxO transcriptional activity and upregulating the expression of E3-ubiquitin ligases [8,9]. Collectively, these mechanisms account for most of myostatin’s activity against post-natal muscle growth. In this regard, myostatin may act as an environmental sensor/signaler of nutritional status (particularly in a low amino acid environment) [10] in synergy with the cellular energy sensor, adenosine monophosphate-activated protein kinase (AMPK) [11], promoting a negative feedback loop that inhibits ribosomal biogenesis and, subsequently, mTOR-dependent protein synthesis [12,13,14].

Myostatin is also a negative regulator of muscle stem “satellite” cell proliferation and differentiation at the G1 to S progression phase of mitosis, which maintains satellite cells in a quiescent state [15]. While strong repressor activity of satellite cell proliferation and differentiation through Smad 2/3 signaling may account for a proportion of myostatin’s role in post-natal muscle growth inhibition, myostatin is probably most influential on the regulation of embryonic muscle progenitors during pre-natal muscle growth where its role remains controversial. Embryonic muscle growth is both hyperplastic and hypertrophic: that is, muscle tissue growth involves both increased myofibre number via the accretion of myoblasts > myotubes > myofibres, followed by their relative diametric and longitudinal growth, which is equally dependent upon motor neuron outgrowth and functional innervation [16]. Usually by 7 years of age, hyperplastic muscle growth ceases and, thereafter, only hypertrophic growth is responsible for increased muscle size [17]—this is achieved through protein synthesis, which is dependent upon the genetic material donated through satellite-cell dependent myotube fusion [18]. Myostatin is strongly expressed in embryonic somites where it appears to modulate the balance between proliferation and differentiation of muscle progenitors during development [19], possibly by sensitizing them to pro-differentiation signals (e.g., Notch signaling [20,21]). In this manner, myostatin helps to set both the finite myofibre number as well as the extent of the satellite cell pool, which dictates the capacity for post-natal growth. This is in direct contrast to its strong repressor activity on post-natal muscle growth. Thus, myostatin apparently exerts very different effects on skeletal muscle growth in the embryonic versus the post-natal cellular environments.

The differential growth of skeletal muscle in the pre- and post-natal environment is important to this story because proof of concept efficacy of myostatin inhibition in animal models of DMD was initially proven through *MSTN* knockout (KO), which manipulates both pre- and post-natal hyperplastic and hypertrophic muscling [22]. This is in stark contrast to drug inhibitors of myostatin, which can only manipulate the post-natal hypertrophic growth of muscle. It might be that myostatin inhibition is much more influential on global muscle mass when applied from the earliest muscle growth during embryogenesis—this explains the phenomenal impact of *MSTN* mutations in mice [1], larger order animals (dogs [23], sheep [24], cows [25], horses [26,27]) and humans [2]. Indeed, in *MSTN* KO mice, hyper-muscling is attributable to hyperplasia (i.e., >80% more muscle fibres) more so than hypertrophy (~30% larger cross-sectional area of the individual muscle fibres [1,28]). Nevertheless, each of the experimental drugs which failed in clinical testing were shown to modify the disease course of murine DMD in the gold standard *mdx* mouse model [29,30,31,32], albeit, only some of these studies used sexually mature mice [29,32] as opposed to juvenile mice [30,31], which are likely to be more amenable to myostatin inhibition [33] through hyperplasia. Wagner has reported that mice (*mdx*) maintain ~10-fold higher circulating myostatin levels than humans and that myostatin repression is ~3-fold higher [4]. This implies that more robust myostatin repressor activity is required to keep muscle mass constrained in mice compared to humans. Thus, human muscle mass may, by comparison, be relatively less modifiable by drugs that interfere with myostatin activation than murine muscle mass. This raises the question: why?

**Figure 1:**
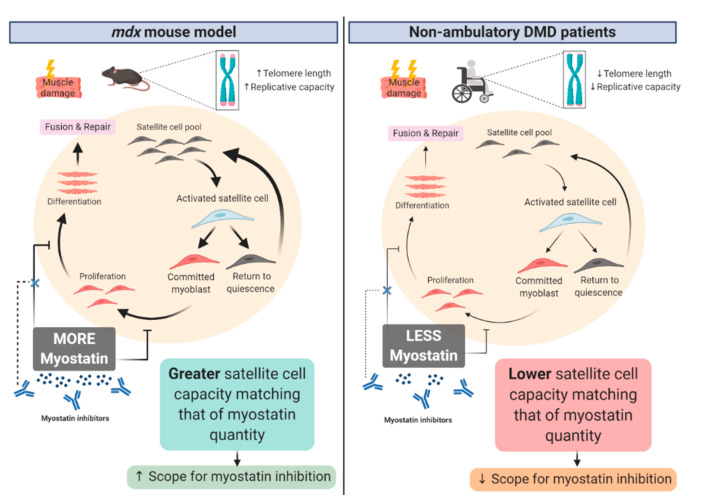
Summary of the contrast between satellite cell regulation in the murine *mdx* model of Duchenne Muscular Dystrophy (DMD) and human DMD patients. It is proposed that a larger and more enduring satellite cell replicative capacity in the *mdx* mouse model of DMD, which is associated with longer telomere length compared to humans, results in a greater scope for myostatin inhibition therapies to prevent myostatin-mediated repression of myoblast differentiation. Conversely, unremitting satellite cell cycling in non-ambulant DMD patients in response to chronic muscle degeneration compared to *mdx* mice, results in depletion of the satellite cell pool, reduced regenerative capacity and, thus, the scope for myostatin inhibition to be therapeutically effective is reduced. Key: ↑ = increased; ↓ = reduced. Created with biorender.com.

Satellite cell regulation of muscle stemness is maintained through daughter cell (muscle progenitor) fate selection, which is under the influence of various factors, including inflammatory cytokines and myokines, growth factors, local cell (i.e., fibro-adipocyte progenitors, extracellular matrix (ECM) and endothelial cells) and non-muscle stem cell signaling, the dystrophin-associated protein complex (DAPC; especially syntrophins), micro and long non-encoding RNA’s, and telomere activity (reviewed in [34]). During moderate-severe muscle damage, satellite cell cycling is amplified, and both daughter cells will commit to symmetric satellite pool expansion initially, before next undergoing asymmetric division where one daughter cell will commit to myogenic lineage and the other to self-renewal of the satellite cell pool to prevent the depletion of stem cell function [35]. This facilitates the rapid repair of lost muscle tissue to maintain mass and function, which is advantageous to the organism during acute muscle injury, but which adversely impacts telomere length. In contrast, during steady-state muscle turnover or minor injury, only asymmetric division occurs [35]. In this scenario, muscle repletion is slower, but telomere shortening is minimized. This is an important concept because muscle regenerative capacity is contingent not only on the number of satellite cells available but also on their proliferative capacity, i.e., how many times they can re-enter the cell cycle. Satellite cells have a finite replicative life which is proportional to telomeric DNA length [36]. It is a well-established pathogenic feature of DMD that the satellite cell pool becomes exhausted (both in satellite cell content and proliferative capacity) due to unremitting cycles of chronic muscle injury and regeneration caused, fundamentally, by the absence of dystrophin protein and alterations to the DAPC. In this setting, satellite cells are less able to exit the cell cycle and revert to their homeostatic quiescent state, which is required for telomere maintenance and long-term regenerative capacity [35]. Compared to humans and for reasons unknown, mice have remarkably longer telomeres, which do not substantially shorten through replication and aging [37]. This suggests a greater comparative capacity for satellite cell activity-mediated regeneration and muscle growth and may explain why the *mdx* mouse recapitulates a milder phenotype than human DMD, particularly since knockdown of telomerase in the *mdx* mouse induces “human-like” DMD [38]. Thus, an exaggerated myostatin repressor function might be fundamentally important to restrain a naturally higher propensity for muscle growth in mice compared to humans because satellite cell activity is more robust and enduring. This effect seems to even endure the elevated demand for muscle regenerative activity caused by dystrophin-deficiency, as suggested by the higher relative suppression of circulating myostatin levels in *mdx* mice (25%) versus DMD patients (8%) compared to healthy controls [4]. For this reason alone, myostatin may be more amenable to inhibition in mice but less so in humans, simply because there is more of it to inhibit and thus the scope for biological modification of muscle growth is greater (as summarized in Figure 1). It would be interesting to determine the capacity for myostatin inhibitor drugs to modify murine DMD using the *mdx*/mTR^-^/^-^ model, which has shortened telomeres, as a staple in pre-clinical trials, where the scope for modification is more comparable to human DMD [39,40].

## 3. Myostatin Inhibition Enhances Muscle Mass but Not Function

**Figure 2:**
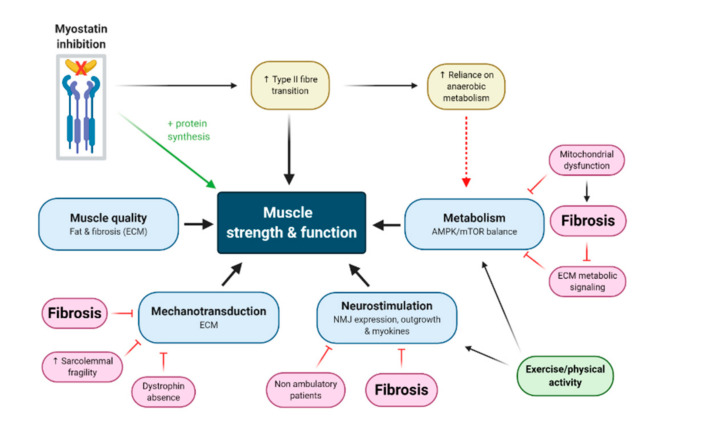
Schematic summarising the factors that may interplay and contribute to lack of functional gains during myostatin inhibition therapy in Duchenne Muscular Dystrophy (DMD) patients. While myostatin inhibition results in increased protein synthesis and muscle accretion, contractile assembly and strength and function gains are driven by a variety of additional factors including composition of the extracellular matrix (ECM), mechanotransduction signaling, neuromotor stimulation and metabolism. Each of these factors are complicated by various aspects of DMD pathogenesis including increased sarcolemmal fragility caused by dystrophin deficiency, fibrosis and steatosis (fatty infiltration) of the extracellular matrix (ECM), downstream mechanotransduction signalling which is impacted by both the ECM and the absence of dystrophin, and mitochondrial dysfunction which is impacted by increased energy demand for satellite cell function and muscle regeneration, the capacity for AMPK activation (which is reduced by myostatin inhibition) as well as the composition of the ECM, amongst other factors. These factors (especially neuromotor stimulation and metabolic signalling) are further impacted by declining physical activity as patients become wheelchair bound. The negative effects of myostatin inhibition on muscle function could be overcome through dual physical activity prescription which facilitates both neuromotor and metabolic stimulation. Key: ↑ = increased; Created with biorender.com.

Despite the lack of translational outcomes for myostatin inhibitors so far, it is important to note that the clinical trials investigating myostatin inhibitors against DMD did not all fail to elicit muscle mass increases. ACE-031 (NCT01099761) and ACE-083 (NCT02927080), domagrozumab (NCT02310763) and RG6206/R07239361 (NCT03039686), all produced mild (generally < 5%), yet statistically significant increases in muscle/lean mass as measured through non-invasive imaging of DMD patients [4,5]. However, what these studies did fail to show were concomitant improvements in strength which should have accompanied these mass gains. Each of these trials failed to meet either their primary or secondary/surrogate outcome measures concerning muscle function [4,5]. This is important when considered in the context of the endpoint and surrogate outcome measures selected, through which drug efficacy is validated. The only successful outcome for patients is the sufficient attenuation of disease course to maintain or improve quality of life. For most of these trials, functional tests to monitor the progression of DMD in the clinic (i.e., the North Star Ambulatory Assessment) were used as outcome measures to evaluate the efficacy of myostatin inhibition against muscle wasting, and therefore strength decline. However, this works off the assumption that myostatin inhibition can induce both mass and function equally, or that mass increases always produce functional/strength improvements. It is well known that muscle strength and size are not increased in concert [41,42] and neither proportionate loss of mass, nor gain of mass arrest, can explain strength decline as humans age [43]. Indeed, studies of myostatin inhibitor drugs against age-related sarcopenic muscle wasting are consistent with data from the DMD clinical trials and support the lack of synergy between mass and strength, as well as the poor translation of murine myostatin inhibition in clinical trials. In this regard, myostatin inhibition causes type II fibre phenotype shifts and changes to metabolism, which in dystrophic muscle that is already compromised structurally (due to extensive fibrosis and fatty infiltrate as well as sarcolemmal instability, which reduces expression of key neuromuscular junction proteins) and bioenergetically (due to increased energy demand for satellite cell activity and muscle regeneration) prevents functional gains (summarised in Figure 2). Here forward, we will discuss each of these aspects.

### 3.1. Myostatin Induces Type II Fibre Type Transformations which Are More Susceptible to MD

In vivo, muscle fibre growth is initiated through an interplay governed by the net positive difference between protein synthesis and degradation, and satellite cell-dependent myotube fusion, but is functionally modulated by neuromotor activation patterns across the neuromuscular junction and mechanical loading [44]. Strength gains are most intensely observed when type II myosin heavy chain isoforms are preferentially expressed (i.e., over slow type I isoforms). Type II fibres have higher power due to faster contraction velocity and larger cross-sectional areas [45], thus greater type II fibre expression leads to bigger fibres capable of higher force outputs and therefore strength. Type II fibres are induced through specific stimuli, namely intense mechanical loading which induces rapid succession action potential transfer across the neuromuscular junction into the t-tubules, and comparable sarcoplasmic reticulum-mediated calcium (Ca^2+^) transients which are typically longer and more concentrated than for temperate mechanical loading [46,47]. Myostatin inhibition induces slow (type I) to fast (type II) fibre transition [48] (Figure 2), and thus should theoretically evoke strength gains in the clinic. To understand why this may not be possible for DMD patients, one must consider the pathogenesis of the disease, which preferentially drives the wasting of type II(x) fibres first [49,50,51]. Dystrophin stabilizes the sarcolemma during muscle contraction and is, thus, particularly important to type II fibres which bear the brunt of the mechanical load. Type II fibres are more prone to damage for this reason alone, but without dystrophin (i.e., in DMD), mechanical damage is intensely exacerbated. As such, converting fibres towards type II, yet maintaining the lack of dystrophin, may simply make them bigger and more susceptible to damage. Further, type II fibres also lack the magnitude of endogenous antioxidant and cytoprotective responsivity of type I fibres [52,53] because they rely less on oxidative metabolism by the mitochondria, which is a principle source of damaging free radicals in cells. Notwithstanding, type II fibres can produce significant amounts of reactive oxygen species (ROS) during explosive, high-intensity activation, which drives xanthine oxidase activity through the degradation of purine nucleotides: the net result is a rapid and intense ROS production [54]. DMD mitochondria are notoriously dysfunctional (Figure 2) and produce appreciably less adenosine triphosphate (ATP) [55,56,57] but more ROS [56,58,59], placing stress on the anaerobic energy systems, which further drives cytosolic ROS production (i.e., through xanthine oxidase, amongst other ROS producing enzymes). DMD muscles, particularly dystrophin-deficient type II fibres, are thus also more prone to oxidative stress-induced damage [51]. For this reason, drugs that mediate fibre type transformations from fast to slow have been suggested as a therapeutic treatment avenue for DMD [60]. Conversely, myostatin inhibition directly contests this idea by promoting expression of the very fibres that are prone to damage and wasting. This might explain why strength gains are less obvious than mass gains, since any muscle mass induced by higher proportions of larger type II fibres likely manifests as a more severe pathology, which limits functionality. It might also explain why, for domagrozumab, muscle volume gains were observed following the first few months of treatment [61], yet at ~12 months, there was no observable difference in muscle volume compared to placebo [62], i.e., the initial muscle gained had preferentially wasted.

### 3.2. Neuromotor Signaling Might Be Fundamentally Required to Convert Mass Gains to Strength Gains

Without proportionate synthesis of contractile proteins as part of the overall protein synthesis rate, muscle mass gains are futile. Indeed, for sarcopenia (the age-related loss of skeletal muscle mass and function), functional decline has been delineated as the most important indicator of this condition [63], i.e., without functional improvement, there is no point to mass increases. Exercise represents the definitive muscle growth stimulus (particularly eccentric resistance exercise [64]) where intrinsic skeletal muscle molecular signaling of hypertrophy (e.g., ROS, metabolites, and mechanical signals) is synergistically activated alongside motor neuron stimulation [65]. While myostatin inhibition may represent an important molecular signal for hypertrophy and hyperplasia, these events may in fact be futile without synergistic motor neuron activation and mechanical signaling [66,67] (Figure 2). To illustrate this point, it is important to highlight that muscle satellite cells represent the only means for post-natal muscle hyperplasia, except for the fact that developing myotubes have no means to access the neural input required to translate new muscle accretion into functional improvements. For this reason, myotubes must, in post-natal muscle, fuse with pre-existing fibres possessing established neuromuscular connections to gain access to neural motor input and become “functional” [18]. Muscle fibre “splitting” is characteristic of DMD muscles [68] and is purportedly caused by the incomplete fusion of satellite cell-mediated myotubes with existing skeletal muscle fibres [69]. The extent to which incomplete fusion might contribute to corresponding increases in function comparative to skeletal muscle accretion is unknown but can be logically interpreted. The less able a myotube is to fully fuse with a given skeletal muscle fibre, the less access its nucleus has to the molecular signals of skeletal muscle contraction, e.g., the t-tubular action potential and the myoplasmic Ca^2+^ and ROS transients among others, which denote the magnitude of mechanical stress relative to neuronal input. A flow on effect of this is a reduction in myokine signals released from muscles during activation, which in turn, attract nerve outgrowth, an essential event that must accompany hypertrophic growth of fibres to maintain proportionate function [70,71,72]. In the muscles of *mdx* mice, aberrant pre- and post-synaptic neuromuscular junction changes have been described [73], but otherwise, *mdx* mice maintain robust contractile function and ambulation throughout life [74]. This might explain why in *mdx* mice, myostatin inhibition can induce both muscle mass and function gains [29,75], although this is not always the case, particularly when considering force production independent of mass gains [76], endurance [76] and as *mdx* mice age [33]. In DMD patients, neuromuscular junction vulnerability is evident [73], which along with reduced neural input through developmental delay of weight-bearing activities such as standing and walking, and wheelchair confinement following loss of ambulation, could contribute significantly to the impaired translation of the neurological signal to skeletal muscles (Figure 2). Combined with myostatin inhibitor drugs, the likely outcome is muscular hypertrophy without the synergistic improvement of skeletal muscle function. To this effect, a novel dual pro-neuromuscular junction and soluble activin receptor myostatin inhibitor protein (ActR-Fc-nLG3), which forsakes the extent of hypertrophy normally shown with pharmacological myostatin inhibition to increase acetycholine receptor surface area at the neuromuscular junction, was shown to improve muscle functional endurance of mice in the rotarod test [77]. As yet, this drug is untested in preclinical animal models of DMD but demonstrates scope for combinatorial therapeutics that target both ACVR2 and neuromuscular junction signalling to elicit dual mass (albeit smaller) and strength gains.

### 3.3. Myostatin Inhibition Interferes with Muscle Metabolism and Endurance

**Figure 3:**
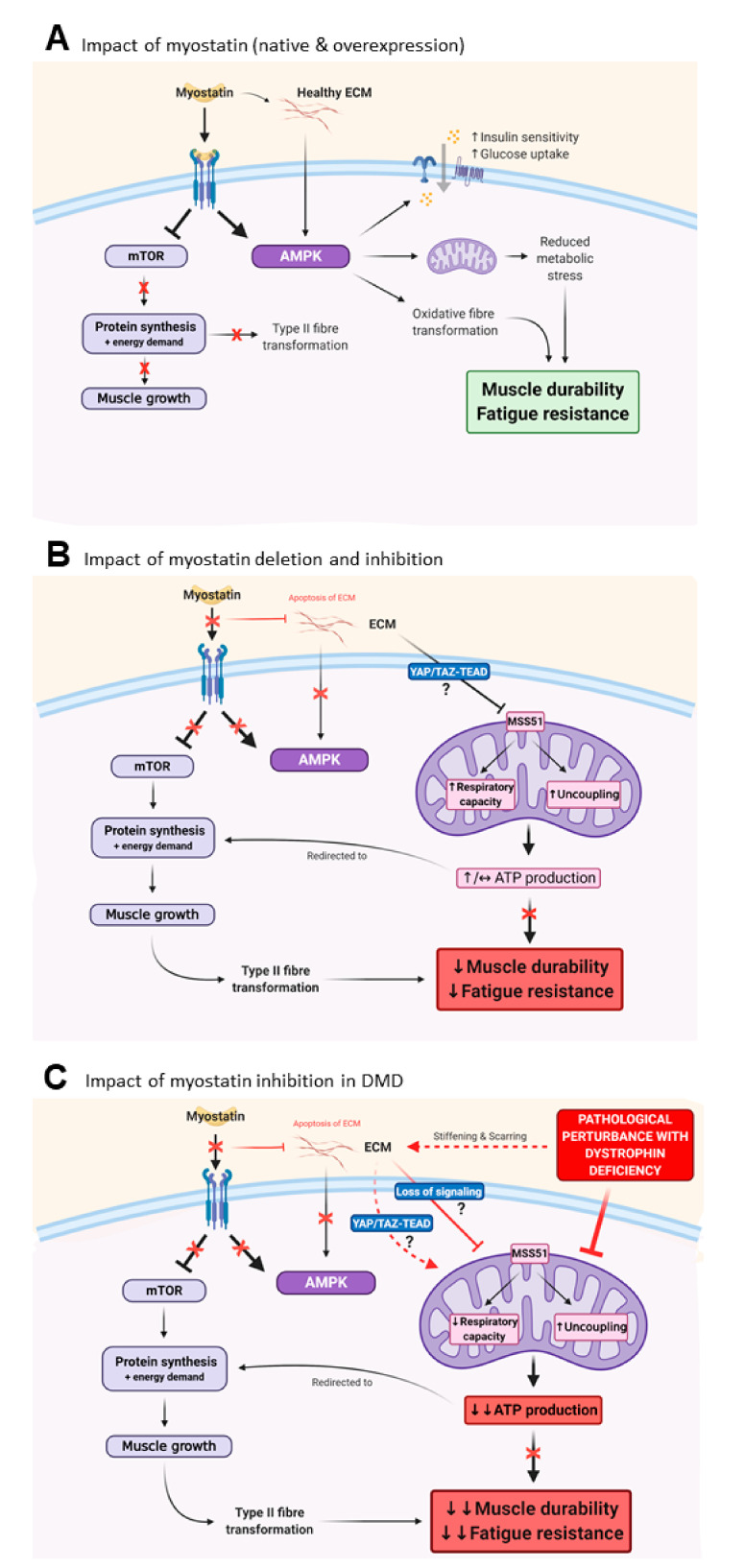
The impact of myostatin and its inhibition on muscle metabolism and functional endurance. (**A**) Native and overexpression of myostatin increases insulin sensitivity and glucose uptake and utilization in skeletal muscle in an adenosine monophosphate-activated protein kinase (AMPK)-dependent manner that is proportional to the extent of myostatin expression. This pro-metabolic function mediated through AMPK is consistent with myostatin’s role as an inhibitor of mammalian target of rapamycin (mTOR)-mediated protein synthesis and muscle growth. (**B**) Myostatin inhibition (pharmacological and genetic) results in changes to metabolic signaling, namely, through removal of myostatin’s repressor activity on mTOR, which results in AMPK inactivation and fibre phenotype shifts toward fast glycolytic type, which reduces oxidative capacity. However, myostatin inhibition also results in secondary inhibition of the mitochondrial regulatory protein, Mss51, possibly through extracellular matrix (ECM) mechanotransduction signaling of YAP-TAZ-TEAD, which are known to promote mitochondrial enzyme activity and respiratory activity through uncoupling. Depending upon the model (i.e., healthy versus fat-fed (obesity) versus hyperglycaemic (diabetic)), the overall effect on mitochondrial energy output can be positive or unchanged, but since energy is directed towards muscle accretion, the overall effect on muscle performance is reduced fatigue resistance and function. (**C**) In dystrophic skeletal muscle, extensive ECM pathology may impact pro-mitochondrial signaling, resulting in reduced respiratory capacity, skeletal muscle fatigue and functional decline, which effectively negates any strength improvements induced through muscle phenotype shifts and accretion by myostatin-inhibition. Key: ↑ = increased; ↓ = reduced; ↓↓ = extremely reduced; ↔ = unchanged Created with biorender.com.

The other important piece of the mass versus function puzzle is the more recently appreciated, yet equivocal, role myostatin plays in regulating muscle metabolism [78,79]. Skeletal muscle functional gains concern not only the ability to produce force (i.e., strength) but the durability of that force output to ensure muscles are relatively fatigue resistant, which is enabled through oxidative metabolism (Figure 2). Both myostatin and its genetic and pharmacological inhibition exert control over metabolism in different ways, and, at least in genetically deleted/mutated loss of functional myostatin animal models, this can result in increased susceptibility to fatigue and reduced muscle function [80,81,82]. Myostatin itself (and its overexpression) increases skeletal muscle insulin sensitivity as well as glucose uptake and utilization through increased glucose transporter expression and glycolysis, alongside inhibition of glycogen synthesis, in cell culture and in healthy mice [78]. These effects are governed through manipulation of AMPK-mTOR balance in favour of AMPK-mediated metabolic homeostasis [78] and are consistent with the inhibition of mTOR-dependent protein synthesis and muscular atrophy controlled by myostatin (Figure 3A). Glucose uptake and utilization are also controlled by the ECM and myostatin may also modulate glucose metabolism indirectly through increasing collagen deposition within the ECM [83] (Figure 3A). Interestingly, in mouse models of obesity and type II diabetes mellitus, genetic and pharmacological inhibition of myostatin also increases insulin sensitivity and glucose uptake, and decreases total body fat [80,81,82]. These effects appear to be via elevated expression of genes regulating fatty acid transport and oxidation, and browning of white fat [82,84] and might also be indirectly controlled through altering the composition of the ECM (i.e., reducing the collagen content of the ECM through myostatin inhibition). Recent studies suggest that myostatin inhibition causes concurrent reductions in mitochondrial regulatory protein, Mss51, resulting in increased cellular ATP levels and mitochondrial respiratory capacity due to increased uncoupling and improved fat metabolism [85,86] (Figure 3B). These data highlight that during myostatin inhibition, a synergistic AMPK-independent pro-metabolism complements the higher energy demand required to facilitate protein synthesis and muscle growth during AMPK inactivation and is possibly driven by the ECM (Figure 3B). As muscle accretes, the effect is an overall higher energy expenditure (i.e., whole body metabolism) and resistance to diet-induced hyperglycaemia and obesity [80,81,82]. Yet controversially, in otherwise healthy *MSTN* KO mice, muscles exhibit increased fatigability during ex vivo [87] and in vivo [88] testing, which is underscored by several mitochondrial anomalies. These include altered enzyme activities [87], DNA depletion [87], uncoupling of respiration [87] and structural alterations including significantly reduced cardiolipin [89], an important mitochondrial membrane phospholipid that controls respiration, energy conversion, ROS production and signalling of mitophagy and apoptosis [90]. Lipid uptake and metabolism is also critically compromised [89,91], including in *mdx* mice following pharmacological myostatin inhibition [92], and may entirely or partly account for the observed mitochondrial dysfunction by limiting the supply of reducing equivalents to sustain contractile activity and therefore muscle function. While the extent of these alterations may be due to the complete absence of myostatin (as in *MSTN* KO models), which drives almost pathological changes to ECM composition and its signalling of metabolism [83], these data are also consistent with the type II fibre type transition that is also driven by pharmacological myostatin inhibition, especially as aerobic exercise training can counter some of these metabolic impairments even though myostatin is genetically deplete [93,94,95]. Exercise can also improve some of the metabolic impairments caused by pharmacological myostatin inhibition in *mdx* mice [92,96,97], highlighting that this repression of metabolism is modifiable and suggesting scope for combinatorial treatment regimens involving exercise training or pharmacological exercise mimetics [96,97]. As an example of the latter, AICAR (5-aminoimidazole-4-carboxamide-1-D-ribofuranoside) has been shown to attenuate oxidative mitochondrial function deficits caused by *MSTN* deletion in mice, but to what extent similar effects could be observed with pharmacological myostatin inhibition has not been investigated yet. Collectively, these studies highlight two fundamental problems with myostatin inhibition: (1) promoting a predominantly fast glycolytic phenotype and extensively altering the ECM to muscle ratio appears ultimately detrimental to muscle function (Figure 3B); and (2) that the regulatory role of myostatin over metabolism is highly dependent upon the genetic and pathophysiological context.

Mitochondrial dysfunction and metabolic stress are well characterised pathogenic features of dystrophin deficient muscles from mice (*mdx* and D2*mdx*) and humans, as extensively documented by us [55,56,57] and others [98,99,100,101,102,103] (for a review, please see [54]). In DMD patients, and even in female carriers of the dystrophin gene mutation [104,105], muscle fatigue and exercise intolerance are reported. For this reason, mitochondrial targeted therapeutics have been tested pre-clinically in *mdx* mice, with encouraging results [56,100,101,106,107,108]. However, akin to myostatin inhibitors, these compounds have not progressed successfully through clinical testing into an effective medicine, as most recently highlighted by the discontinuation of the clinical development program of the coenzyme Q10 analogue, idebenone, by Santhera Pharmaceuticals [109]. It might be that in the chronic and progressive pathogenic environment of human dystrophin deficient muscles, metabolism cannot be modified enough to improve functional endpoint measures—at least through the targets which have been investigated thus far. Given the role of ECM composition in the regulation of metabolism, this may well be due to the extensive fibrosis observed as DMD progresses in patients (discussed in detail in Section 3.3; Figure 3C). This is in contrast to *mdx* mouse muscles, which, while also exhibiting mitochondrial and metabolic dysfunction, manifest a relatively milder disease course until older age (i.e., >12 months age) [110] and therefore less metabolic demand/stress and fibrosis compared to DMD patients. Due to the high cost associated with experimenting on older mice with progressive MD, pre-clinical testing of myostatin inhibitor drugs has never included them. Indeed, in a severe Limb Girdle Muscular Dystrophy R1 mouse model (C3KO) that also manifests progressive muscle loss in concert with extensive fibrosis and mitochondrial perturbations consistent with human DMD patients, genetic (*follistatin*) and pharmacological (anti-myostatin) inhibition of myostatin leads to significant muscle mass gains yet no improvement in strength nor endurance, due to reduced AMPK signalling and consequential loss of oxidative capacity [111].

### 3.4. Fibrosis of the Extracellular Matrix (ECM) May Drive Unmodifiable Loss of Function

Mechanotransduction occurs through a complex interplay between bone, connective tissue and muscle signalling to ensure muscle and bone growth are synchronous and that tendons are strong enough to facilitate the forces that are exerted on the connection [112]. In addition to the repression of muscle growth, myostatin exerts control over bone morphogenic proteins (BMPs [5]; especially BMP2) and fibroblasts to stimulate bone resorption and the synthesis of ECM proteins such as collagen [113], respectively. One reason for myostatin’s strong repressor function on muscle and bone growth in the face of strong pro-fibrogenic activity might be that tendons grow much slower than either muscle or bone, thus through transiently repressing muscle and bone growth, tendon strengthening can “catch up” to guarantee stability of the bone-tendon-muscle unit [112]. Genetic myostatin inhibition (i.e., through deletion or polymorphisms) results in mismatched muscle and bone growth compared to tendon and ECM development, which in higher order animals (cattle and dogs, including the GRippet (golden retriever muscular dystrophy [GRMD] crossed with the myostatin-deficient Whippet) canine model of DMD) results in joint instability and the reduced mass of many organs including lung and spleen [23,114,115]. In *MSTN* KO mice, functional decline is evident from young age and extends into middle age as measured through grip strength and treadmill exercise, and this coincides with significant ankle joint pathology involving bone, tendon, articular connective tissue and synovial fluid anomalies with inflammatory infiltrate [116]. The long-term consequences of such changes are unclear for pharmacological myostatin inhibitors but could ultimately be debilitating, particularly for DMD patients who already experience significant deterioration of respiratory function as disease progresses due to fibrosis of the diaphragm and accessory respiratory muscles.

Pharmacological and genetic myostatin inhibition not only reduces fibrogenesis, but also induces apoptosis of existing ECM [4]. Thus, for pathologies underscored by uncontrolled fibrosis, such as DMD, this aspect should be beneficial rather than deleterious. Pathological fibrosis is a significant contributor to loss of muscle strength and function in human DMD muscle [117,118,119], yet is relatively less severe in *mdx* muscles except for diaphragm [120]. Fibrosis is driven by chronic pro-inflammatory signalling of fibroblasts to secrete collagen and fibronectin caused by uncontrolled muscle damage and wound healing processes [118]. Many studies have shown that fibrosis of dystrophin deficient *mdx* skeletal muscles can be reduced through pharmacological myostatin inhibition [22,30,113,121,122], yet surprisingly, muscle quality (i.e., muscle:fat:fibrosis) was never assessed in any of the myostatin inhibitor clinical trials in DMD patients. Thus, whether fibrosis could be modified in human DMD patients as per in *mdx* mice remains unknown—indeed, if it were, it was not to an extent that afforded functional improvements. A recent clinical trial testing ginvinostat (ITF2357), a potent histone deacetylase activity inhibitor known to reduce fibrosis and enhance muscle regeneration similar to myostatin inhibitors, biopsied patients and showed a significantly increased muscle fraction alongside reduced fibrotic tissue [123]. However, improved functional testing was not observed in this study either, suggesting that reducing fibrosis alone cannot impact the loss of strength and function associated with disease progression in humans (although it should be acknowledged that this study was reported as underpowered [123]). Importantly, the composition of the ECM exerts regulatory control over metabolism and contraction through complex mechanotransduction signalling pathways [83]. With the loss of dystrophin, a key mechanotransduction protein, and significant ECM alterations caused by chronic inflammation that results in scarring and stiffening, muscle fibres become disconnected from the ECM, resulting in loss of mechnotransduction signalling, and subsequently metabolic and functional decline [83]. Because *mdx* mice manifest less ECM fibrosis compared to human patients, allowing muscle fibres to maintain better connection with the ECM, they may be more amenable to metabolic and functional modification imposed through anti-myostatin actions on the ECM. Thus, the pre-existing degree of muscle fibrosis should be an important consideration in determining inclusion/exclusion and subject stratification criteria during clinical trial design.

## 4. Corticosteroids Interfere with Myostatin Inhibition

In pre-clinical animal studies, it is relatively simple to control for the many confounding variables that can inadvertently impact research outcomes. This becomes more difficult in clinical trials with humans, and ever more difficult again with rare diseases when there are so few patients to access. Corticosteroids (prednisolone/prednisone, or deflazacort) are standard of care for DMD treatment (which may also include angiotensin converting enzyme (ACE) inhibitors or angiotensin blockers) [124], but due to significant side-effects, not all DMD patients are amenable to them. This leaves a very small population of DMD boys who are steroid naïve and prime candidates for trialing candidate therapeutics. For the rest of the DMD patient population, standard of care cannot be ethically withdrawn, which has important implications for trial participant selection and stratification. Inevitably, there is a trade-off that must be made between having a sufficiently powered trial to establish efficacy of a therapeutic candidate, which can only be achieved with larger numbers of study participants, and introducing significant confounders to the research.

Paradoxical to their use against muscle wasting, corticosteroids are atrophic agents, but were introduced for the treatment of DMD due to their potent anti-inflammatory and immuno-modulatory action [124]. There has been only one myostatin inhibitor drug tested pre-clinically in combination with corticosteroids. Hammers et al. demonstrated that when administered with prednisone, the muscle mass increases induced by myostatin inhibition (with a pro-peptide) were abolished [125]. Importantly, the failed trials testing domagrozumab and RG6202 enlisted patients receiving the standard of care, giving scope for drug interactions which likely impacted myostatin inhibition capacity.

**Figure 4:**
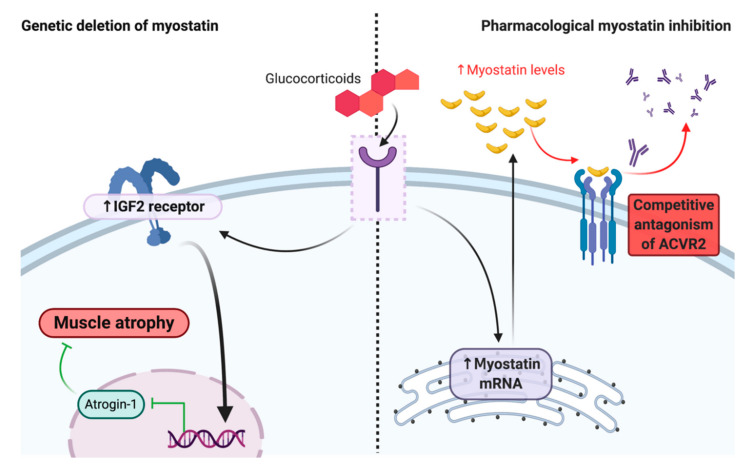
Basic schematic depicting the molecular response to combined glucocorticoid treatment with myostatin deletion and pharmacological inhibition. In myostatin knock-out mice, the atrophic effects of glucocorticoid therapy are mitigated through up-regulation of the insulin-like growth factor 2 (IGF2) receptor, which represses Atrogin-1 transcription and muscle atrophy. This effect is not apparent with pharmacological myostatin inhibition that targets the activin receptor 2 (ACVR2) because myostatin transcription is still biologically active. Rather, glucocorticoids increase myostatin mRNA expression, which could elevate circulating myostatin levels to such an extent that they competitively antagonize pharmacological myostatin inhibitor binding of the ACVR2 receptor. Key: ↑ = increased. Created with biorender.com.

Genetic myostatin deletion can curb the muscular atrophy effects of corticosteroid treatment, suggesting that myostatin may modulate corticosteroid receptor signaling in skeletal muscle [28]. In healthy mouse muscle, glucocorticoids reduce IGF-1 mRNA, leading to the removal of IGF-R1 repression of atrogin-1, and thus causing muscle atrophy. When administered to *MSTN* KO mice, the same skeletal muscles respond to corticosteroid treatment by upregulating IGF-2, which can alternatively interact with the IGF-R1 receptor [28] to repress atrogin-1 and muscular atrophy (Figure 4). While these data ostensibly suggest that myostatin inhibition could be useful to counteract the side effects of corticosteroid treatment, i.e., muscle atrophy, it is important to highlight that corticosteroids also increase the expression of myostatin mRNA and protein [126,127,128] (Figure 4). Since this effect cannot occur in genetically ablated KO mice, but can during drug-induced myostatin inhibition where the *MSTN* gene is functional, it stands to reason that corticosteroids may directly oppose myostatin inhibitor drug action through increasing myostatin levels to competitively antagonize the ACVR2 or myostatin activation. In this regard, whether a DMD clinical trial participant is steroid treated (as opposed to steroid naïve), and then specifically, the dosage of corticosteroid administered to individual participants, would be highly influential on the capacity of myostatin inhibitor drugs.

## 5. Conclusions

Myostatin inhibitor drugs have the potential to be greatly beneficial against muscle wasting diseases and disorders, yet to date, have been highly ineffective. The dramatic impact of loss of function myostatin mutations on muscle mass and strength accretion, which are probably most profoundly influential during embryonic development, must be balanced against the capacity of drugs to resist skeletal muscle wasting driven by a plethora of stimuli in the post-natal environment. The complex environment of diseased and degrading muscle presents an even more difficult landscape for pharmacological myostatin inhibitors to contend with. Clinical trials in DMD patients present a variety of challenges which make participant stratification, and the selection of primary and secondary outcome measures, difficult, and confounding variables numerous. We suggest that in addition to corticosteroid use as standard of care, the physical capacity of patients as well as their relative level (volume and intensity) of physical activity should be considered when testing myostatin inhibitors, since neural, mechanical and metabolic input is likely very impactful on eliciting functional improvement alongside mass gains. Emerging evidence suggests that myostatin not only regulates muscle mass, but also metabolism, adiposity and insulin-sensitivity [129]. Pursuing downstream molecular targets of myostatin rather than upstream activation and receptor binding could thus represent an alternative druggable target against DMD. For example, targeting Mss51, which provides positive metabolic effects without the deleterious fast glycolytic phenotype transitions and AMPK inactivation associated with myostatin inhibition, could be more beneficial for the treatment of DMD. Alternatively, combinatorial regimens involving pharmacological myostatin inhibitors with exercise or drugs that amplify the mechanical and/or metabolic signal associated with exercise, or alternatively, gene delivery or exon skipping therapeutics designed to re-establish some degree of dystrophin protein expression back into dystrophic muscles [130,131], could bring together the benefits of both treatments to produce better translational outcomes for DMD patients.
